# Food to some, poison to others ‐ honeybee royal jelly and its growth inhibiting effect on European Foulbrood bacteria

**DOI:** 10.1002/mbo3.397

**Published:** 2016-10-14

**Authors:** Thomas V. Vezeteu, Otilia Bobiş, Robin F. A. Moritz, Anja Buttstedt

**Affiliations:** ^1^ Institut für BiologieZoologie – Molekulare ÖkologieMartin‐Luther‐Universität Halle‐WittenbergHalle (Saale)Germany; ^2^ Departamentul de Apiculturǎ şi SericiculturǎFacultatea de Zootehnie şi BiotehnologiiUniversitatea de Ştiinţe Agricole şi Medicinǎ VeterinarǎCluj‐NapocaRomania

**Keywords:** antibiotic, *Apis*, immunity, *Melissococcus*, MRJP

## Abstract

Honeybee colonies (*Apis mellifera*) serve as attractive hosts for a variety of pathogens providing optimal temperatures, humidity, and an abundance of food. Thus, honeybees have to deal with pathogens throughout their lives and, even as larvae they are affected by severe brood diseases like the European Foulbrood caused by *Melissococcus plutonius*. Accordingly, it is highly adaptive that larval food jelly contains antibiotic compounds. However, although food jelly is primarily consumed by bee larvae, studies investigating the antibiotic effects of this jelly have largely concentrated on bacterial human diseases. In this study, we show that royal jelly fed to queen larvae and added to the jelly of drone and worker larvae, inhibits not only the growth of European Foulbrood‐associated bacteria but also its causative agent *M. plutonius*. This effect is shown to be caused by the main protein (major royal jelly protein 1) of royal jelly.

## 1 Introduction

Adult honeybees (*Apis mellifera*) feed their growing larvae in the hive with different food jellies depending on sex, caste, and age. Royal larvae receive only royal jelly (RJ), a secretion of the hypopharyngeal and mandibular glands of young worker bees, comprising all the nutrients required to develop into a queen (Snodgrass, 1925). Worker and drone larvae are fed with RJ supplemented with honey and pollen obtained from the stores within the hive. This huge amount of food stored at optimal temperatures (~30°C) and humidity (~60%) for bacterial and fungal growth provide ideal conditions for the growth of all kinds of pathogens. Given that every larva in the colony gets its share of the RJ pie, it may not be surprising that RJ harbors antibiotic properties against a variety of bacteria (Fujiwara et al., 1990; Hinglais, Hinglais, Gautherie, & Langlade, 1955; McCleskey & Melampy, 1939), though the vast majority of the studies performed are linked to bacteria causing human diseases. When it comes to bee pathogens, amazingly little is known about the effect of RJ on honeybee‐specific pathogens given the scientific and public awareness for global colony losses (Moritz & Erler, 2016; Potts et al., 2010). Most research has been confined to the gram‐positive bacterium *Paenibacillus larvae* responsible for the brood disease American Foulbrood (AFB) (Erler & Moritz, 2016; White, 1906).

However, colonies can also die from European foulbrood (EFB), another brood disease associated with a variety of different bacteria (Forsgren, 2010). Although the main trigger for EFB is the gram‐positive microaerophilic *Melissococcus plutonius* (Forsgren, 2010; White, 1912) other bacteria, for example, *Enterococcus faecalis*,* Paenibacillus alvei*,* Brevibacillus laterosporus*,* Bacillus pumilus*, and *Achromobacter euridice*, have been shown to co‐occur with EFB as the so‐called secondary invaders (Erler, Denner, Bobiş, Forsgren, & Moritz, 2014; Forsgren, 2010). Whereas, *M. plutonius* is undoubtedly pathogenic and triggers EFB, *P. alvei*,* B. pumilus*, and *A. euridice* have also been identified as common bacterial species of the adult honeybee intestinal microflora (Gilliam, 1997) and *B. laterosporus* was detected in honeybee larvae, pupae, and foragers without being pathogenic (Marche, Mura, & Ruiu, 2016). However, besides the co‐occurrence with *M. plutonius*, the role of the secondary invaders during EFB progress is not understood.

We are only aware of three studies on the antibiotic effect of RJ on the EFB‐associated bacteria *P. alvei* (Lavie, 1960) and *E. faecalis* (Garcia, Finola, & Marioli, 2013; Sauerwald, Polster, Bengsch, Niessen, & Vogel, 1998) with Pierre Lavie indicating that this effect is not only attributable to the fatty acid 10‐hydroxy‐2‐decenoic acid (10‐HDA) (Lavie, 1960), which had previously been identified as an antibiotic compound (Blum, Novak, & Taber, 1959; Garcia et al., 2013; Yousefi et al., 2012). It has been shown that one mode by which 10‐HDA interferes with bacteria is by inhibiting the biosynthesis of glucan polymers, which are critical for the adherence of *Streptococcus mutans* to eukaryotic cell surfaces (Yousefi et al., 2012). In addition to 10‐HDA, also the protein components of RJ have been investigated for antibacterial properties. The classical antimicrobial peptide defensin has also been identified in RJ although it was originally called royalisin (Fujiwara et al., 1990). However, the honeybee defensin is not one of the main proteins of RJ, these are made up of the major royal jelly proteins (MRJPs) accounting for approximately 80% of total RJ proteins (Buttstedt, Moritz, & Erler, 2014; Schmitzová et al., 1998). Although *mrjps* are expressed in all body sections of all castes of the honeybee, the focus of the expression of *mrjp1* to *4* lies clearly in the heads of nurse bees that house the RJ‐producing food glands (Buttstedt, Moritz, & Erler, 2013). Whereas it has been shown that MRJP2 exhibits antibacterial activity (Bíliková et al., 2009; Feng et al., 2015), the antibacterial potential of the main protein of RJ, MRJP1, cannot be assessed with certainty with some publications describing an antibacterial effect (Brudzynski & Sjaarda, 2015; Brudzynski, Sjaarda, & Lannigan, 2015) while others do not (Bucekova & Majtan, 2016; Feng et al., 2015). Nevertheless, MRJP1 is thought to be a precursor of the jelleins, short (~ 1 kDa) antimicrobial peptides derived from the C‐terminus of MRJP1 also present in the larval food (Fontana et al., 2004). However, the majority of the bacteria tested in these studies were not linked to honey bee diseases and nothing is known about the antimicrobial activity of MRJPs against *M. plutonius* and secondary invaders of the honeybee disease EFB. Here we report on the effect of RJ and its main protein MRJP1 on *M. plutonius* and on bacterial species associated with EFB, which may reveal evolutionary relevant adaptations rather than screening human pathogens.

## 2 Material and Methods

### 2.1 Bacterial strains and cultivation

The gram‐positive bacterial strains *M. plutonius* (LMG 20360, biological origin: honeybee larvae), *E. faecalis* (LMG 7937), *P. alvei* (LMG 13253, biological origin: foul brood of honeybees) and *B. laterosporus* (LMG 16000, biological origin: soil and water) were obtained from the BCCM/LMG Bacteria Collection (Ghent University, Ghent, Belgium). *B. pumilus* (SLU 119‐12) was isolated from honeybee larvae showing EFB symptoms (Erler et al., 2014). *Escherichia coli* and *Pseudomonas fluorescens* were used to test the effect of RJ on gram‐negative bacteria. The respective media were as follows: 5 g/L peptone, 3 g/L meat extract, pH 7.0 for *P. alvei*,* B. laterosporus* and *P. fluorescens*; 5 g/L yeast extract, 10 g/L tryptone, 5 g/L NaCl for *E. coli*; 10 g/L Müller‐Hinton broth, 15 g/L yeast extract, 8 g/L peptone from casein, 3 g/L peptone from soymeal, 3 g/L KH_2_PO_4_, 2 g/L glucose, 1 g/L sodium pyruvate, pH 7.2 for *E. faecalis* and 10 g/L glucose, 7.5 g/L peptone, 6.8 g/L KH_2_PO_4_, 2.5 g/L yeast extract, 2 g/L tryptone, 2 g/L starch, pH 7.2 for *B. pumilus*. After autoclaving the latter medium was further supplemented with L‐cysteine at a concentration of 250 mg/L for *M. plutonius*. For agar plates, 1.5 g/L agar agar was added. All bacteria, except *M. plutonius*, were first cultivated at 37°C on agar plates from glycerin cultures stored at −80°C. A single colony was chosen to generate a streak plate that was stored at 4°C and served as inoculum for overnight cultures. *M. plutonius* was cultivated at 37°C in 15 ml liquid medium in 15 ml conical tubes, directly inoculated from the glycerin culture, in a carbon dioxide incubator (10% CO_2_) (UniEquip, Planegg, Germany) to ensure a microaerophilic environment during the first cultivation step.

### 2.2 Bacterial growth assays

Bacterial growth assays were performed following the 96‐well plate protocol of (Erler et al., 2014), which allows for monitoring of the whole bacterial growth phase and aims at the determination of the lag phase length and of the slope during the logarithmic phase. Briefly, for *E*. *faecalis*,* B. pumilus*,* P. alvei*,* B. laterosporus, E. coli*, and *P. fluorescens* fresh overnight cultures in liquid media were used to inoculate 200 μl medium per well in a 96‐well plate (Greiner Bio‐One, Kremsmünster, Austria) at an optical density (OD_600 nm_) of 0.001. The plates were incubated under medium shaking for 24 hr at 37°C in a Synergy Mx microplate reader (BioTek, Winooski, VT, USA) and the OD_600 nm_ was measured every 15 min. *M. plutonius* failed to grow under these conditions and we modified the technique with an inoculation at an OD_600 nm_ of 0.1 and incubation without shaking at 37°C for 72 hr. All treatments were measured in at least five replicates. Preliminary tests revealed that the direct addition of pure RJ interfered with OD measurement as the absorbance at 600 nm was too high to measure any bacterial growth even with just 5% RJ. Thus, we decided to use RJ water extracts at concentrations of 2–10%. In addition, a sugar control solution (0.03 g/ml glucose, 0.03 g/ml fructose, 0.005 g/ml sucrose) was analyzed at the same dilutions as for RJ water extracts to exclude the possibility that the antibiotic effect could be attributed to the osmotic effect of sugar. To test for the antibiotic effect of MRJP1, purified protein was added at final concentrations between 15 and 500 μg/ml to the respective media. Growth curves were fitted after subtraction of the medium control with CurveExpert Professional 2.2.0 to a modified Logistic model using y(t) = A/(1 + exp((4μ/A)×(λ−t)+2)) with μ  =  maximum slope; λ  =  length of lag phase; A  =  maximum cell growth and t  =  time. Growth inhibition (I) has been determined based on the slopes of the RJ water extract bacterial growth curves (μ_WE_) in relation to sugar or media control growth curves (μ_C_) with I  =  (μ_C_−μ_WE_)/μ_C_.

### 2.3 Royal jelly samples

Royal jelly (RJ) samples were purchased from Naturprodukte Lembcke GbR (Faulenrost, Germany) (RJNP, imported from China), Cum Natura GmbH (Bühl, Germany) (RJCUM, imported from Thailand) and Aspermühle Naturwaren‐Niederrhein GmbH (Goch‐Asperden, Germany) (RJASP, imported from China) and frozen directly after arrival at −20°C in 2 g aliquots. None of the samples showed any antibiotic contamination above the detection limit (analyses performed by Intertek Holding Deutschland GmbH, Leinefelden‐Echterdingen, Germany). The antibiotics tested were streptomycin, sulfonamides, and trimethoprim for all RJ samples as well as tetracyclines, chloramphenicol, nitrofurane metabolites, nitroimidazoles, fluoroquinolones, and macrolides for RJNP and RJCUM.

### 2.4 Preparation and analysis of royal jelly water/media extracts

To prepare the RJ water/media extracts, 2 g of RJ were either mixed with 2 ml double distilled water for the physicochemical analysis or with 2 ml of the respective medium for the bacterial growth assay. Samples were centrifuged twice for 10 min at 20,000*g* to ensure the removal of any solids. Glucose, fructose, and sucrose contents were determined by HPLC (Sesta, 2006) using a Shimadzu VP series liquid chromatograph equipped with a degasser, pump, controller, column oven and an auto injector (Shimadzu Scientific Instruments, Columbia, USA). Acetonitril/water 75% (v/v) was used as the mobile phase for the separation of sugars on an Alltima Amino 100Å 5 μm column (256 × 4.6 mm) at a flow rate of 1.3 ml/min, 30°C and detected with an RID‐10A refractive index detector. The 10‐HDA content was determined according to (Liu, Yang, Shi, & Peng, 2008) with a Shimadzu VP series liquid chromatograph, photo diode array detector, and an LC‐18 (5 μm) column (256 × 4.6 mm) with LC‐18 2CM precolumn KIT. Total protein content was determined using the Bradford (Bradford, 1976) method and BSA (bovine serum albumin) as the standard for calibration curve. Measurements at 595 nm were made on a Shimadzu UV Spectrophotometer.

### 2.5 Purification of MRJP1 from RJ

1 g RJ was homogenized in 20 mmol/L sodium citrate, pH 4.0 (buffer 1) to a total volume of 10 ml. The solution was centrifuged at 8500*g* for 10 min at 4°C, the supernatant dialysed against buffer 1 (Spectra/Por^®^ 6 Dialysis Membrane MWCO: 25 kDa, Spectrum Laboratories, Rancho Dominguez, CA, USA), to remove any low molecular weight compounds, for example, sugars, and again centrifuged at 20,000*g* for 10 min at 4°C. The supernatant was loaded onto a 1.5 ml column containing Sulphopropyl (SP) Sepharose Fast Flow (GE Healthcare, Little Chalfont, UK) and MRJP1 was found to be in the flow through (Fig. S1). All other proteins bound to the column and were eluted with buffer 1 containing 1 mol/L NaCl. The antibacterial protein defensin, with a pI of 8.64, was strongly positively charged at the pH of 4.0 and thus bound to the column. In preceding experiments, it turned out that the buffer used during purification (20 mmol/L sodium citrate, pH 4.0) reduced the growth of *Escherichia coli* (data not shown) and thus MRJP1 was dialysed against double distilled water (Spectra/Por^®^ 6 Dialysis Membrane MWCO: 25 kDa, Spectrum Laboratories, Rancho Dominguez, CA, USA) prior to the growth assay experiments. After dialysis, the protein concentration of MRJP1 in double distilled water was determined as 1.4 mg/ml according to (Bradford, 1976) using BSA as standard (standard 2 mg/ml, ThermoFisher Scientific, Waltham, MA, USA) and thus 18.2 mg pure MRJP1 could be obtained from 1 g RJ. Protein purity was confirmed with the help of sodium dodecyl sulfate (SDS) polyacrylamide gel electrophoresis (PAGE) (Laemmli, 1970) (Fig. S1). The protein solution was initially diluted 1:1 with double concentrated media, for example, 10 g/L yeast extract, 20 g/L tryptone, 10 g/L NaCl for *E. coli*, to maintain concentrations of the media ingredients before further dilution with normal media in the growth assays.

The RJ water extracts contained on average 46 mg/ml total proteins of which 30% are thought to be MRJP1 (Schmitzová et al., 1998) and thus the extracts contained about 14 mg MRJP1 per ml. Therefore, we decided to use MRJP1 in a first experiment at a concentration of 500 μg/ml approximately reflecting the concentration of the protein in the 4% RJ water extracts.

### 2.6 Statistics

Statistical analyses were performed with STATISTICA 8.0 (StatSoft, Tulsa, OK, USA). To determine the impact of the sugar control, slopes of bacterial growth curves were compared using one‐way analysis of variance (ANOVA) followed by post hoc Scheffe test if data met the criteria for normal distribution according to Kolmogorov–Smirnov or by Kruskal–Wallis ANOVA if not normally distributed. The influence of the three different RJ samples on the growth of *E. faecalis*,* B. pumilus*,* P*. *alvei* and *B. laterosporus* were analyzed using a general linearized model (GLM) (log link function and gamma distribution) with transformed data (growth inhibition + 0.06) to eliminate negative data points. The effect of 500 μg/ml MRJP1 on EFB bacteria was determined by pairwise comparisons of the growth curve slopes with and without MRJP1 using the Mann–Whitney U test (MWU). Dose–response experiments were analyzed with one‐way ANOVA followed by post hoc Scheffe test. Data for *B. pumilus*,* E. coli*, and *P. fluorescens* were normally distributed, whereas data points collected for *E. faecalis* were transformed according to the following equation f(x) = exp(x).

## 3 Results

### 3.1 Physicochemical analysis of royal jelly water extracts

Approximately half of the amounts of sugars found on average in pure RJ were regained in the water extracts with 3.44% glucose compared to 6.83%, 2.98% fructose compared to 5.85% and 0.45% sucrose compared to 1.09%, all by weight (Table SI). The extracts contained 4.62% total proteins compared to 11.71% in RJ. The fatty acid 10‐HDA showed only a marginal solubility in water (0.23% compared to 1.70% in RJ). Values for commercial RJ samples are adopted from (Pavel et al., 2014).

### 3.2 Effect of royal jelly water extracts on bacterial growth

To determine the impact of RJ on the growth of the different bacterial strains, growth curves were recorded in standard medium and supplemented with sugar solution or RJ water extracts. The sugar control did not influence the growth of *E. faecalis* (ANOVA, MS = 0.006, *F* = 3.842, *p *≥* *.080), did enhance the growth of *B. pumilus* at the highest sugar concentration (Kruskal–Wallis ANOVA, *H* = 14.843, *p *≤* *.03); and that of *B. laterosporus* (ANOVA, MS = 0.001, *F* = 7.527, *p *≤* *.02), and *P. alvei* at all sugar concentrations measured (ANOVA, MS = 0.007, *F* = 13.292, *p *≤* *.02) (Table S2). Thus, the strong inhibitory osmotic effect of sugar on bacterial growth found in honey (Erler et al., 2014) does not apply at the relatively low sugar concentrations in the RJ water extracts.

**Figure 1:**
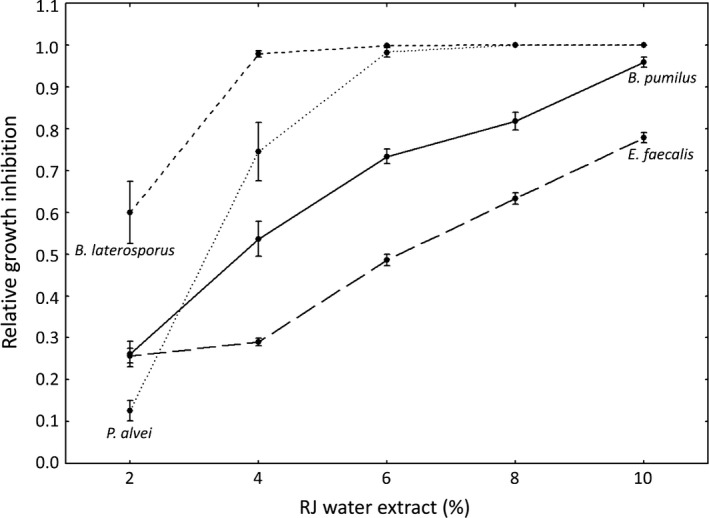
Relative growth inhibition in dependence of RJ water extract percentage averaged over all three RJ types (means ± standard errors (SE)). Growth inhibition was determined using the slopes of the bacterial growth curves. *B. pumilus* – solid line; *E. faecalis* – long dashed line; *P. alvei* – dotted line, *B. laterosporus* – short dashed line

The extracts decelerated the growth of all four EFB‐associated bacteria, by decreasing the slope and extending the lag phase of the growth in a concentration‐dependent manner (Table S2). Due to the microaerophilic nature of *M. plutonius* its growth was barely detectable in the positive control. The addition of any concentration of RJ water extracts completely prevented any bacterial growth (Table S2). Since *M. plutonius* growth was only observed in the positive control under different settings (OD measurements for 72 hr without shaking) than for the other bacteria, it was excluded from statistical analyses of the other bacteria, using a GLM. Growth inhibition in relation to the sugar control was determined based on growth curve slopes during the log phases (Fig. 1). Bacterial species, percentage of the RJ water extract and the interaction between both had a tremendous effect on the inhibition of bacterial growth (GLM; bacteria: *W* = 366.228, *df* = 3, *p *<* *.0001; RJ percentage: *W* = 1269.534, *df* = 4, *p *<* *.0001; bacteria × RJ percentage: *W* = 324.730, *df* = 12, *p *<* *.0001), whereas the type of RJ and the interaction between the RJ type and percentage did not have any impact (RJ type: *W* = 1.960, *df* = 2, *p *=* *.375; RJ type × percentage: *W* = 11.177, *df* = 8, *p *=* *.192).

### 3.3 Effect of MRJP1

**Figure 2:**
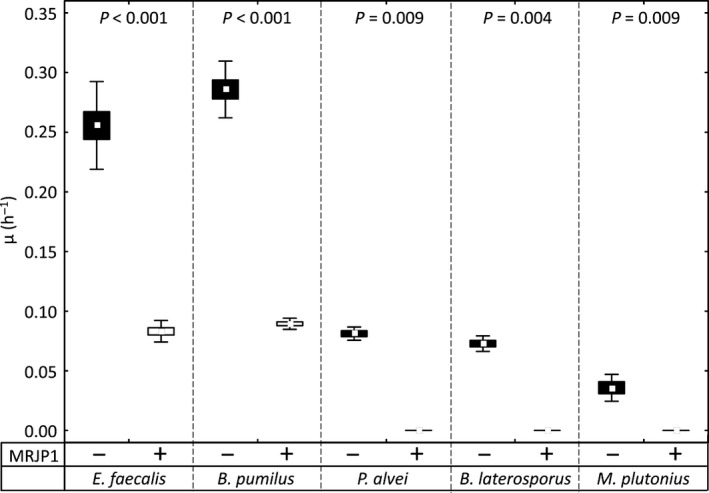
Growth curve slopes (μ) of *E. faecalis*,* B. pumilus*,* P. alvei*,* B. laterosporus*, and *M. plutonius* in medium without (black boxes) and with (white boxes) the addition of 500 μg/ml MRJP1 (means ± SE and standard deviation (SD))

The addition of MRJP1 (500 μg/ml) significantly reduced the slopes for all bacterial growth curves measured (Fig. 2; MWU, Z ≥ 2.611, *p *≤* *.009) with the growth completely inhibited for *P. alvei*,* B. laterosporus*, and *M. plutonius* and a growth inhibition compared to the positive control of 0.67 ± 0.04 for *E. faecalis* and 0.69 ± 0.02 for *B. pumilus*.

**Figure 3:**
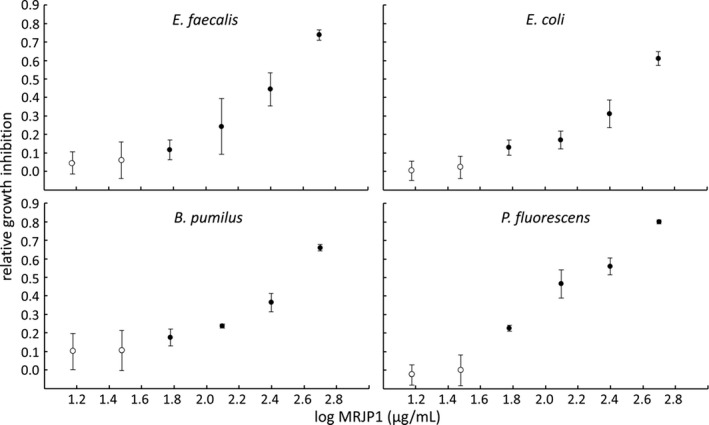
Relative growth inhibition (means ± SD) of 15–500 μg/mL MRJP1 on the growth of *E. faecalis*,* B. pumilus*,* E. coli*, and *P. fluorescens*. Closed circles represent values significantly different from the positive control without MRJP1 (One‐way analysis of variance (ANOVA), post hoc Scheffe test, *p *≤* *.012)

To see if the effect of MRJP1 was concentration dependent, serial dilution experiments with 500–15 μg MRJP1 per ml medium were performed with the rapidly growing gram‐positive bacteria *E. faecalis* and *B. pumilus*. In addition, experiments were also conducted with the gram‐negative species *Escherichia coli* and *Pseudomonas fluorescens*. Although these bacteria are not associated with any bee disease, they shed light on the effect of MRJP1 on gram‐negative bacteria in general. Whereas 15 and 30 μg/ml did not have an effect, 60 μg/ml significantly decelerated the growth of all four bacterial species tested (ANOVAs, MS = 0.03–0.37, *F* = 48.86–188.69; Scheffe test *p *≤* *.012) and 500 μg/ml showed a relative growth inhibition between 0.61 (*E. coli*) and 0.80 (*P. fluorescens*) (Figure 3).

## 4 Discussion

Here we show for the first time that RJ decelerates the growth of *M. plutonius* the causative agent of EFB and of the secondary invaders of the disease, that is, *E. faecalis*,* B. pumilus*,* P. alvei* and *B. laterosporus*. The addition of 2% RJ water extract did already completely inhibit the growth of *M. plutonius*, whereas the secondary invaders *B. laterosporus* and *P. alvei* were able to grow, albeit impaired, in the presence of low amounts of RJ water extracts (<6%). Finally, *E. faecalis* and *B. pumilus* grew slowly even in the presence of 10% RJ water extract, the highest concentration tested in this study. However, the bacterial species showed already remarkable different growth curves in their recommended media without RJ with *E. faecalis* and *B. subtilis* exhibiting a much better growth than *B. laterosporus*,* P. alvei* and *M. plutonius* (Table S2).

In contrast to honey, where different honey types may be pathogen specific (Erler et al., 2014; Gherman et al., 2014), the effect of RJ seems to be independent of origin. Furthermore, we illustrate that a large proportion of the antibiotic effect of RJ can be attributed to MRJP1. The purified protein seems to be slightly more effective than the RJ water extract, especially for *E. faecalis* where the 4% RJ water extract, containing approximately 500 μg/ml MRJP1, showed a relative growth inhibition of only 0.29 compared to 0.67 with purified MRJP1 at the same concentration. However, RJ supplies a variety of nutrients to the growing larvae and contains in addition to antibacterial substances, for example, 10‐HDA, defensin, MRJP1, and MRJP2, also sugars, free amino acids, and vitamins which accelerate bacterial growth. Furthermore, whereas on the one hand a protein might act as an antibiotic, it can also serve as a nutrient for the same bacterial species after degradation by extracellular proteases known to be produced by pathogens, for example, the AFB causing bacterium *P. larvae* (Antúnez, Arredondo, Anido, & Zunino, 2011). Thus, the individual influence of each ingredient and their potential interactions adding up to the overall effect of RJ on bacterial growth is at best difficult to disentangle in the light of the complex composition of RJ.

Whereas honeybee defensin has been shown to only affect gram‐positive bacteria (Fujiwara et al., 1990), 10‐HDA inhibits both, gram‐negative and gram‐positive bacteria but only at comparatively high concentrations (minimum inhibitory concentration: 0.5–2 mg/ml for gram‐positive; not determined for gram‐negative bacteria) (Blum et al., 1959; Garcia et al., 2013; Yousefi et al., 2012). Thus, the relatively low amounts of 10‐HDA in the 2–10% RJ water extracts (0.05–0.23 mg 10‐HDA/ml) do not add to the antibiotic effect in this study. MRJP1 starts to inhibit the growth of both gram‐type bacteria significantly at the concentration of 60 μg/ml. This concentration is approximately 580 times lower than the concentration of MRJP1 in pure RJ, given an average protein content of 11.71% and an MRJP1 amount of 30% (~35 mg MRJP1/g RJ) (Pavel et al., 2014; Schmitzová et al., 1998).

However, our results contrast to two other reports, who did not detect any antibacterial effect of MRJP1 (Bucekova & Majtan, 2016; Feng et al., 2015). Indeed, Bucekova and Majtan tested the antibiotic effect of MRJP1 up to a concentration of 47.5 μg/ml well below the minimal inhibitory concentration of 60 μg/ml found in our experiments. Unfortunately, Feng et al. (2015) do not specify MRJP1 concentrations. Furthermore, both studies only report the end‐point measurements after an incubation of 18 hr or 24 hr, respectively (Bucekova & Majtan, 2016; Feng et al., 2015) not allowing for analyzing the temporal growth dynamics over time. Following the growth curves over a period of 24/72 hr, we show that the addition of up to 500 μg/ml MRJP1 indeed decelerates the growth of *E. faecalis*,* B. pumilus*,* E. coli*, and *P. fluorescens* by decreasing the slopes of the growth curves but not the end‐point measurements. Only those bacteria that already showed poor growth in normal medium (*P. alvei*,* B. laterosporus* and *M. plutonius*) did not start to grow in the presence of 500 μg/ml MRJP1 over the measured time period.

Brudzynski and Sjaarda (2015) and Brudzynski et al. (2015) found an antibacterial effect of MRJP1 with minimal inhibitory concentrations between 5 and 33 μg/ml using end‐point measurements. However, in these studies, the proteins had been isolated from honey by Concanavilin A affinity chromatography which does not lead to pure MRJP1, but instead to the isolation of glycosylated proteins. Indeed, SDS‐PA gels showed a variety of bands in addition to the one of MRJP1 (Brudzynski & Sjaarda, 2015). We therefore agree with these authors that the activity of additional proteins in the honey might have contributed to the antimicrobial properties. For example, short antimicrobial peptides derived from the C‐terminus of MRJP1 (jelleins) have been shown to act as antibiotics at low concentrations (2.5–30 μg/ml) (Fontana et al., 2004).

Here, we show that in addition to MRJP2 (Bíliková et al., 2009; Feng et al., 2015), also full length MRJP1 adds to the overall antibiotic effect of RJ by decelerating the growth of all bacteria tested. Given the high concentrations of MRJP1 in honeybee food jelly, this effect might still be sufficient to protect the majority of the larvae from bacterial diseases. But how does MRJP1 interfere with bacterial growth? It has already been described that RJ and most likely the highly glycosylated MRJPs therein block the lectin‐mediated adherence of *Pseudomonas aeruginosa* to target cells (Lerrer, Zinger‐Yosovich, Avrahami, & Gilboa‐Garber, 2007). Furthermore, the antibacterial activity of MRJP2 depends on the type of glycosylation (Bíliková et al., 2009; Feng et al., 2015) and the glycosylation of MRJPs isolated from honey mediates the agglutination of *E. coli* and *B. subtilis* cells (Brudzynski & Sjaarda, 2015). Thus, glycosylations of MRJPs seem to be involved in growth inhibition by affecting cell–cell interactions. During the early infection process of EFB, *M. plutonius* is located along the peritrophic membrane of the midgut of the growing larvae, and multiplies as the disease advances to finally fill the entire lumen of the intestine (Takamatsu, Sato, & Yoshiyama, 2016; White, 1920). Hence, MRJP1 might impede cell–cell interactions that are mandatory for progression of EFB. However, compared to AFB, where it is clear that *P. larvae* actively destroys the peritrophic membrane, adheres to the subjacent midgut epithelial cells, and subsequently invades the hemocoel (Garcia‐Gonzalez & Genersch, 2013; Poppinga et al., 2012), EFB is a bee disease where this kind of detail has not been investigated and thus any attempt to explain the effect of MRJP1 remains speculative. It has only recently been shown that the peritrophic membrane degenerates after *M. plutonius* infection but the cells do not invade the larval body cavity (Takamatsu et al., 2016). Compared to the severe etiopathology induced by *P. larvae*, this may appear not to be particularly dramatic. However, the integrity of the peritrophic membrane is crucial for larval survival (Garcia‐Gonzalez & Genersch, 2013) which may be why *M. plutonius* infections result in larval death.

Given the variety of antibacterial substances in RJ, it may seem impossible that bacteria can provoke diseases at all in the presence of RJ. However, RJ is not always supplied in equal and certainly not always in sufficient amounts. In *M. plutonius‐*infected colonies, most larvae die when the larvae nurse ratio increases and the amount of food jelly provided becomes insufficient for infected larvae (Bailey, 1983). Thus, the EFB infection might only cause larval death if the amount of food jelly received is unusually low. Both larval malnutrition and an underrepresentation of antibacterial substances may allow *M. plutonius* cells in the midgut to reach critical quantities causing larval death.

## Funding Information

This project was supported by the German Research Foundation (Deutsche Forschungsgemeinschaft – DFG, grant MO 373/32‐1 to RFAM) and an ERASMUS + exchange program grant to TVV. The sponsors had no role in study design; in the collection, analysis, and interpretation of data; in the writing of the report and in the decision to submit the article for publication.

## Conflict of Interests

None declared.
