# Supplementary material for: Food to some, poison to others ‐ honeybee royal jelly and its growth inhibiting effect on European Foulbrood bacteria
Source: Microbiologyopen. 2016 Oct 14;6(1):e00397. doi: 10.1002/mbo3.397 (PMC5300887; doi:10.1002/mbo3.397)
Supplement: Supplementary file 1 [file MBO3-6-0-s001.pdf]

SUPPLEMENTARY INFORMATION

Food to some, poison to others - honeybee royal jelly and its growth inhibiting effect on European Foulbrood bacteria

Thomas V. Vezeteu, Otilia Bobiş, Robin F. A. Moritz & Anja Buttstedt

---

Figure SI: 10% SDS-polyacrylamide-gel showing purified MRJP1.

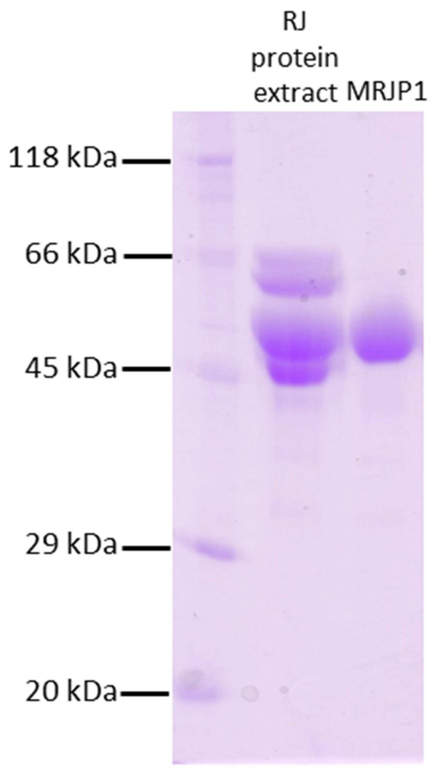

Table SI: Fructose, glucose, sucrose, protein and 10-HDA content of the royal jelly water extracts (RJWE) with all values given in percent (*w*/*w*).

RJ WE	10-HDA	Fructose	Glucose	Sucrose	Protein
RJNP	0.22	2.99	3.87	0.25	5.15
RJASP	0.23	3.08	3.28	0.62	4.62
RJCUM	0.23	2.88	3.17	0.48	4.08
mean ± SD	0.23±0.01	2.98±0.10	3.44±0.38	0.45±0.19	4.62±0.54

RJNP: royal jelly purchased from Naturprodukte Lembcke GbR; RJASP: royal jelly purchased from Aspermühle Naturwaren-Niederrhein GmbH; RJCUM: royal jelly purchased from Cum Natura GmbH; SD: standard deviation; 10-HDA: 10-hydroxy-2-decenoic acid.

Table SII: Influence of RJ water extracts (WE) on maximal slopes (μ) and lengths of lag phases (λ) for growth curves of indicated bacterial species. Values are given as means ± standard deviation.

	WE	μ (h^{-1})				λ (h)			
	(%)	RJNP	RJCUM	RJASP	sugar	RJNP	RJCUM	RJASP	sugar
E. faecalis	0	0.44±0.06				3.70±0.11			
	2	0.29±0.01	0.29±0.01	0.33±0.03	0.41±0.03	3.99±0.04	3.89±0.03	4.02±0.04	3.43±0.12
	4	0.26±0.01	0.26±0.01	0.27±0.02	0.37±0.03	4.37±0.05	4.13±0.04	4.85±0.07	3.43±0.05
	6	0.18±0.02	0.20±0.01	0.18±0.02	0.36±0.02	5.11±0.13	4.60±0.07	4.40±0.24	3.49±0.08
	8	0.14±0.01	0.15±0.02	0.12±0.01	0.38±0.04	4.54±0.11	5.19±0.22	4.93±0.40	3.47±0.09
	10	0.11±0.01	0.09±0.01	0.07±0.01	0.41±0.03	6.51±0.32	5.24±0.12	5.68±0.75	3.59±0.06
B. pumilus	0	0.33±0.02				3.44±0.20			
	2	0.29±0.03	0.22±0.01	0.22±0.01	0.33±0.02	3.96±0.06	4.00±0.04	4.03±0.14	3.28±0.05
	4	0.21±0.02	0.13±0.01	0.10±0.01	0.32±0.02	4.81±0.46	5.20±0.10	5.81±0.30	3.22±0.07
	6	0.12±0.02	0.08±0.01	0.07±0.01	0.34±0.01	8.43±0.58	6.50±0.06	9.08±1.48	3.44±0.15
	8	0.06±0.03	0.03±0.00	0.04±0.01	0.33±0.01	14.68±0.74	7.56±0.23	11.66±0.78	3.24±0.02
	10	0.03±0.03	0.03±0.02	†	0.45±0.01	19.31±4.40	12.66±6.36	†	3.44±0.07
P. alvei	0	0.07±0.01				5.70±0.43			
	2	0.15±0.02	0.14±0.01	0.14±0.01	0.17±0.03	21.89±0.36	5.90±0.09	17.62±0.35	6.94±0.36
	4	0.01±0.00	0.08±0.01	0.02±0.02	0.14±0.03	22.25±3.91	8.17±0.10	14.38±9.19	6.69±0.37
	6	†	†	†	0.12±0.03	†	†	†	6.07±0.51
	8	†	†	†	0.15±0.01	†	†	†	6.63±0.33
	10	†	†	†	n.d.	†	†	†	n.d.
B.laterosporus	0	0.07±0.01				5.52±0.16			
	2	0.01±0.00	0.06±0.00	0.06±0.01	0.10±0.01	22.25±3.91	5.49±0.03	10.44±0.76	5.74±0.23
	4	†	0.01±0.00	†	0.11±0.02	†	5.55±1.06	†	5.79±0.25
	6	†	†	†	0.09±0.01	†	†	†	6.06±0.10
	8	†	†	†	0.10±0.01	†	†	†	6.39±0.16
	10	†	†	†	n.d.	†	†	†	n.d.
M. plutonius	0	0.04±0.01				30.76±14.35			
	2	†	†	†	n.d.	†	†	†	n.d.
	4	†	†	†	n.d.	†	†	†	n.d.
	6	†	†	†	n.d.	†	†	†	n.d.
	8	n.d.	n.d.	n.d.	n.d.	n.d.	n.d.	n.d.	n.d.
	10	n.d.	n.d.	n.d.	n.d.	n.d.	n.d.	n.d.	n.d.

Grey shaded boxes highlight for the slope (μ) percent of inhibition compared to sugar control; light grey - 26 to 50% inhibition, middle grey - 51 to 75% inhibition, dark grey - 76 to 100% inhibition; and for the length of the lag phase (λ) percent of lag phase extension compared to sugar control; light grey - 26 to 50% extension, middle grey - 51 to 75% extension, dark grey - more than 76% extension. † - no bacterial growth detectable within 24 (*E.faecalis*, *B. pumilus*, *P. alvei* and *B. laterosporus*) or 72 h (*M. plutonius*); n.d.: not determined; RJNP: royal jelly purchased from Naturprodukte Lembcke GbR; RJASP: royal jelly purchased from Aspermühle Naturwaren-Niederrhein GmbH; RJCUM: royal jelly purchased from Cum Natura GmbH. Statistical analysis with a general linearized model (GLM) using transformed data (growth inhibition + 0.06) revealed a significant effect on growth inhibition by the bacterial species, percentage of RJ water extract and the interaction between both (bacteria: W = 366.228, df = 3, $P < 0.0001$; RJ percentage: W = 1269.534, df = 4, $P < 0.0001$; bacteria × RJ percentage: W = 324.730, df = 12, $P < 0.0001$).
